# Protective Biomolecular Mechanisms of Glutathione Sodium Salt in Ischemia-Reperfusion Injury in Patients with Acute Coronary Syndrome-ST-Elevation Myocardial Infarction

**DOI:** 10.3390/cells11243964

**Published:** 2022-12-08

**Authors:** Alessio Arrivi, Francesco Barillà, Roberto Carnevale, Martina Sordi, Giacomo Pucci, Gaetano Tanzilli, Francesca Romana Prandi, Enrico Mangieri

**Affiliations:** 1 Interventional Cardiology Unit, Santa Maria University Hospital, 05100 Terni, Italy; 2 Department of Systems Medicine, Tor Vergata University of Rome, 00133 Rome, Italy; 3 Department of Medical-Surgical Sciences and Biotechnologies, Sapienza University, 04100 Latina, Italy; 4 Mediterranea Cardiocentro, 80122 Napoli, Italy; 5 Unit of Internal Medicine, S. Maria University Hospital, 05100 Terni, Italy; 6 Department of Medicine and Surgery, University of Perugia, 06123 Perugia, Italy; 7 Department of Clinical, Internal Medicine, Anesthesiology and Cardiovascular Sciences, Sapienza University of Rome, 00161 Rome, Italy; 8 Department of Cardiology, Mount Sinai Hospital, Icahn School of Medicine at Mount Sinai, New York, NY 10029, USA

**Keywords:** glutathione, STEMI, primary PCI, angioplasty, reperfusion injury

## Abstract

Ischemia-Reperfusion Injury (IRI) is responsible for adverse outcomes in patients with ST-Elevation Myocardial Infarction (STEMI). Oxidative stress, resulting from the production of Reactive Oxygen Species (ROS) and low availability of Glutathione (GSH), are the two main mediators of IRI. The effectiveness of exogenous antioxidant therapy in this scenario is still debated, since the encouraging results obtained in animal models have not been fully reproduced in clinical studies. In this review we focus on the role of GSH, specifically on the biomolecular mechanisms that preserve myocardial cells from damage due to reperfusion. In this regard, we provide an extensive discussion about GSH intrinsic antioxidant properties, its current applications in clinical practice, and the future perspectives.

## 1. Introduction

In the last 40 years, medical research in the field of ischemic heart disease has mostly followed a dichotomous trend, from ischemia to reperfusion. Specifically, in the early 1980s research focused on “Ischemia” and its treatment, first with fibrinolytic therapy [1] and then with primary percutaneous coronary intervention [2,3,4] (pPCI). The effectiveness of pPCI in the ST-Elevation Myocardial Infarction (STEMI) setting has been well established [5], currently representing the gold standard reperfusion technique [6]. However, the early advantage of the restored coronary artery patency is amended by the so called “Ischemia-Reperfusion Injury” (IRI), which is responsible for adverse outcomes [5,7,8]. To date, IRI is still a concern, since we do not have a well-established therapy yet [9].

## 2. STEMI and IRI: A Misbalance between ROS Activity and GSH Availability

**Figure 1:**
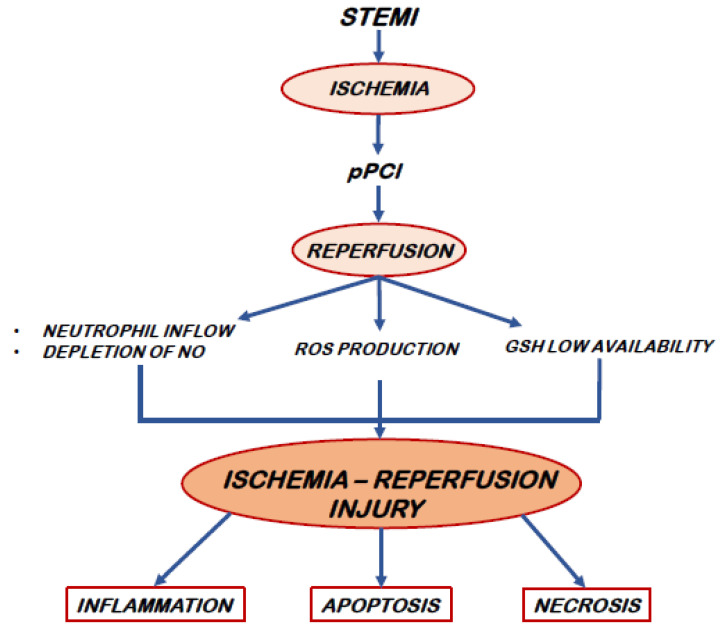
The pathophysiology of IRI. Abbreviations. STEMI: ST-elevation myocardial infarction, pPCI: primary PCI, NO: nitric oxide, ROS: Reactive Oxygen Species, and GSH: glutathione.

Despite timely adequate reperfusion strategies, STEMI-related mortality is currently high (6–12%), as it is the prevalence of heart failure hospitalizations within one year (14–36%) [10]. Myocardial damage resulting from reperfusion and the subsequent death of cardiomyocytes largely explain these adverse outcomes. The experimental evidence from both animal models and clinical investigations suggests that IRI accounts for up to 50% of the final myocardial scar extension [11,12]. Cellular calcium overload and oxidative stress with production of ROS are the main mediators of IRI [9]. The sources of ROS are essentially three: mitochondrial electron transport chain, xanthine oxidase (derived from endothelial cells) and reduced Nicotinamide Adenine Dinucleotide Phosphate (NADPH) oxidase (from activated neutrophils) [9,11,12]. ROS induce the opening of Mitochondrial Permeability Transition Pore (MPTP) that, in turn, results in necrosis and apoptosis of cardiomyocytes. Moreover, ROS also plays a chemotactic action on white blood cells [13,14,15]. In particular, the oxidative burst promotes a long lasting free-radical activity that induces a sustained inflammatory response with a significant increase in leukocytes adherence in the reperfused areas and further massive leukocyte infiltration into the injured myocardium [16,17]. The optimal healing requires a sequential and coordinated recruitment of cells that remove necrotic tissue and improve tissue repair, thereby hindering the expansion of the infarcted area [18,19]. The early phase is characterized by massive neutrophils infiltration [20,21] and additional damage on myocardial cells provoked by neutrophils themselves as a primary source of ROS [22]. Thereafter, monocytes and their descendants’ macrophages dominate the cellular infiltration, promoting an effective tissue regeneration and resolution of the inflammation [23]. Experimental investigations revealed that a misbalance of these sequential phases, as a consequence of an expanded systemic supply of inflammatory cells, could affect infarct healing, leading to adverse outcomes [16]. In this scenario, lymphocytes play a pivotal role by modulating innate immune cell recruitment to the infarcted myocardium. In a mice experimental model of myocardial infarction, CD4+ T lymphocytes cell deficiency delays the monocyte transition and impairs the healing of the heart [24]. Furthermore, an enhanced metabolic activity accompanying T cells activation drives increased production of ROS, that requires anti-oxidative GSH up-regulation to prevent cellular damage [25]. Upon early T cell activation, GSH tissue content allows adequate scavenging activity. The subsequent burst of oxidative status linked to massive lymphocytes proliferation overwhelms GSH anti-oxidant potential affecting T cell growth and differentiation [26,27]. An adequate level of GSH is therefore of paramount importance for the maintenance of an appropriate cell redox environment, thus avoiding or repairing oxidative modifications that involve cell function and survival [28]. Consequently, a reduced availability of this endogenous antioxidant during the early phase of myocardial reperfusion, negatively impacts the cellular repair process, facilitating ROS-mediated myocytic damage [29] [Figure 1]. However, ROS can also be protective as signal preconditioning defense and induce stress responses that guide it to survival [30]. It is possible that cells are largely protected by antioxidants while preconditioning and adaptation signals are prevalent in tissues next to the most ischemic portion. The latter may be injured by addition of antioxidants which hindering in adaptive natural cardio-protection [30]. Thus, the correct balance between ROS production and antagonization is essential for the maintenance of cellular homeostasis.

## 3. Antioxidant Agents in IRI Protection: The Unfinished Controversy

To date, the use of exogenous antioxidant therapy in IRI is still debated. Despite encouraging results obtained from animal models, these benefits have not been fully reproduced in the clinical scenario, leaving uncertainties about its implementation in clinical practice [31]. The reason of inconclusive data probably arises from differences in the use of the various antioxidants (often alone), dissimilarities of drug dosage, time and method of administration between studies [32]. Indeed, for antioxidants to be successful, the agent must be delivered in an appropriate time window (as soon as possible), be able to enter the target tissue and cells, and directed to the sensitive intracellular compartment (e.g., mitochondria and/or lysosomes) [30]. Obviously having all three of these faculties at the same time is very difficult. This would explain the reduced success rate of antioxidant therapy in the everyday medical practice. Antioxidants are classified as hydrophilic, such as ascorbic acid (vitamin C) with ROS scavenger activity, and hydrophobic, such as vitamin E. The latter, as a membrane-bound product, protects against lipid peroxidation [33]. Ascorbate scavenging is a dose-dependent phenomenon and requires intravenous administration to react with superoxide anion radicals, as its plasma concentration is strictly regulated by dose-related excretion [32]. Its administration before elective coronary angioplasty was associated with a reduction of oxidative stress and an improvement in reperfusion parameters [34]. However, the evaluation of clinical outcomes in the setting of IRI remain inconclusive [32]. Similarly, observational and epidemiological data regarding vitamin E in reducing myocardial reperfusion injury are contradictory. Furthermore, its adequate administration on top of the oxidative burden can be beneficial [35], although high doses (>400 IU/d) have been related to an excess of all-cause mortality [36]. The results of N-Acetylcysteine (NAC) administration for cardio-protection are similarly inconclusive [37]. Its acts mainly as GSH-donor. Some studies in the setting of pPCI highlighted a decrease in oxidative stress, with no differences in infarct size [37,38]. A better preservation of the left ventricular function and a decrease in biomarkers of oxidative stress were found when NAC infusion was associated with nitroglycerine and streptokinase [39]. Side effects related to intravenous administration are not to be underestimated, especially after initial loading. Indeed, serious adverse effects, such as anaphylactoid reactions, despite uncommon, were found in up to 8.2% of patients [40]. In addition, iron-chelators, such as deferoxamine, have been tested before and after angioplasty in patients with AMI but were ineffective in determining significant differences in scar extension vs controls [41]. Even preconditioning, postconditioning and remote ischemic conditioning (RIC) studies gave inconclusive results [11,42]. Indeed, the unpredictability of STEMI makes the use of preconditioning difficult in this context, although it may play a role in planned cardiac surgery [43,44]. Whether RIC can actually improve clinical outcomes following pPCI is currently unknown, despite initial promising results before hospital admission in patients with AMI [45]. Therefore, in spite of at least four decades of research, results concerning the use of antioxidant as well as pre/post conditioning therapeutic strategies in limiting IRI are still elusive.

## 4. Antioxidant Properties of GSH

**Figure 2:**
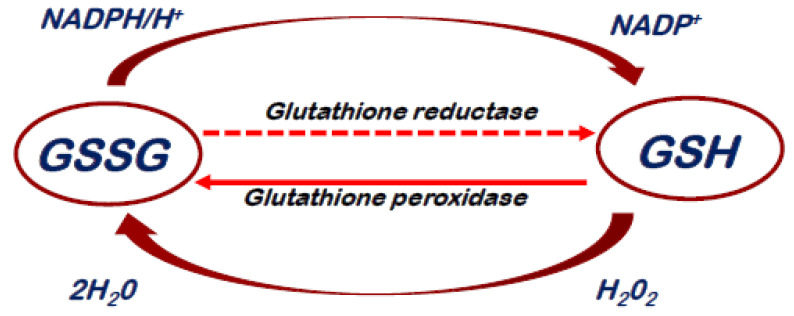
Glutathione Redox cycle. Abbreviations. GSSG: Oxidized Glutathione, GSH: Reduced Glutathione.

GSH is a water-soluble tripeptide. Its chemical structure is composed by a combination of three single amino acids: cysteine, glycine and glutamine [28]. GSH is present in all mammalian cells and plays a role in transport of amino acids, maintenance of the sulfhydryl groups of proteins and protection against oxidizing agents. Its antioxidant power is linked to the sulfur chemical group, a potent reducing agent [28]. GSH is the principal intracellular antioxidant that acts directly by scavenging reactive oxygen and nitrogen species or indirectly by supporting enzymatic activity as a cofactor [46,47]. Glutathione S-transferases and peroxidases catalyze the conjugation of the reduced form of GSH to xenobiotic substrates for the purpose of detoxification [28]. The isoenzymes are cytosolic, mitochondrial and microsomal. The oxidized state (GSSG) could be chemically reverted through the isoenzyme glutathione reductase and NADPH [Figure 2]. An increased GSSG-to-reduced GSH ratio is indicative of oxidative stress [28]. The total cellular GSH content and the GSH/GSSG ratio are controlled by a GSH-negative feedback loop, in response to fluctuating oxidative stress levels [48]. The intracellular and extracellular GSH concentrations are regulated by the balance between its synthesis and catabolism, as well as by its transport between the cytosol and the different organelles or the extracellular area [47]. Of note, GSH could pass through the innermost layer of the mitochondrial membrane and accumulate into the endoplasmic reticulum [49,50]. Much of GSH synthetized in cells is exported across the plasma membrane into the extracellular spaces, especially during oxidative conditions [51]. The synthesis of GSH from glutamate, cysteine, and glycine is catalyzed by two enzymes, those being γ-glutamylcysteine synthetase (GCS) and GSH synthetase [52]. This process is regulated primarily by GCS activity, cysteine availability and GSH feedback inhibition. This pathway occurs in virtually all cell types, with the liver being the major producer and exporter of GSH [52]. Both animal and human studies demonstrated that adequate protein nutrition was determined for the maintenance of GSH homeostasis. Most of acute events that lead to oxidative and/or inflammatory burst increase GCS transcription or activity in a variety of cells [52].

## 5. Biomolecular Mechanisms of GSH Protection against IRI: From Experimental Studies to Clinical Practice

In acute coronary syndrome ST-elevation myocardial infarction (ACS-STEMI), ROS-mediated myocytic cell damage starts in the first minutes of acute reoxygenation (secondary to reperfusion) and lasts for weeks or months through the activation of apoptosis and autophagy processes [53,54]. Among ROS, Hydrogen Peroxide (H_2_O_2_) is produced by many enzymes including xanthine oxidase, lipoxygenase and, specifically, NADPH oxidase [55]. H_2_O_2_ plays an important role in the pathogenesis of myocardial reperfusion injury. Indeed, exposure of cultured human cardiomyocytes to H_2_O_2_ has been shown to determine the rapid onset and progressive oxidative cell death [56]. The antioxidant capacity of GSH is related to its ability to donate hydrogen atoms from its thiol group to most carbon-, oxygen-, and nitrogen-centered radicals [57]. H_2_O_2_ is removed by GSH, a reaction catalyzed by GSH peroxidase, a selenium-dependent enzyme [58]. GSH is then regenerated from glutathione disulfide (GSSG) by GSH reductase, a reaction using NADPH as a cofactor. This redox cycle is important for the cell to combat oxidant stress. Furthermore, GSH is able to limit damage to the lipid membrane by inhibiting lipid peroxidation [59] and can be effective against some highly reactive species such as hydroxyl radical [60,61] and singlet oxygen [54]. As already mentioned, a reduction of the myocardial content of GSH has been observed during ischemia and reperfusion of the ischemic myocardium [62] and it is associated with future cardiac events [63]. This results in a reduction in cellular defenses against the heightened oxidative burden. Animal studies [62] have shown that an increase in myocardial GSH through GSH supplementation is beneficial in protecting against reperfusion injury. They observed a significantly smaller infarct size compared to animals with lower myocardial GSH [62]. Preclinical study of ischemia/reperfusion showed that timely application of GSH provides better cardio-protection at higher doses [64]. Tanzilli et al. demonstrated, within the context of a randomized clinical trial [29,65], that an early (immediately before pPCI) and prolonged (up to 72 h after angioplasty) administration of glutathione sodium salt in patients with STEMI was able to significantly reduce H_2_O_2_ production and decrease isoprostanes serum levels. GSH infusion also promoted early and sustained increase of serum H_2_O_2_ breakdown activity (HBA) and NO bioavailability [65], which was highly related to progressive significant reduction of serological markers of myocardial injury. In addition, the authors showed a progressive significant decrease of serum cardiac Troponin T release during the 5 days of reperfusion in the GSH-treated patients compared with the control group, resulting in a 21% reduction of myocardial damage [65]. Specifically, prophylactic and prolonged GSH infusion was able to reduce NADPH oxidase 2 activation and modulate inflammatory effector cell response, as assessed by changes of differential leukocytes count and TNF-α production over the first 5 days after STEMI [29]. In treated patients, the balance of innate and adaptive immune response determined an improvement of left ventricular function at follow-up [29] and a significant decrease in the hospital length of stay [66]. Based on these results, it could be hypothesized that direct GSH infusion, through both activation of endogenous antioxidant protection mechanisms and attenuation of inflammatory cell recruitment, was able to preserve vital myocardial components and endothelial cell function against a harmful pro-oxidant and inflammatory environment [29].

## 6. Future Perspectives 

A targeted antioxidant therapy that does not interfere with other signaling pathways (such as preconditioning ones), and is readily available for use is a medical goal that must be pursued. The easy availability, low cost and the absence of interactions or relevant side effects [67] make GSH a good candidate for its widespread and safe use for the protection of IRI. Moreover, GSH supplementation during STEMI does not exclude the use other antioxidants such as ascorbic acid that demonstrated beneficial effects in contrast-induced nephropathy [68]. Indeed, acute renal failure following STEMI results from oxidative stress, vasoconstriction and hypoperfusion due to left ventricular dysfunction [69]. Therefore, the scavenging action of the ascorbic acid, clearly demonstrated in contrast-induced nephropathy [68], could be enhanced by an increase in NO bioavailability resulting from the GSH supplementation [65,70]. Based on previous results [29,34,65], it is plausible that a combination of the two molecules applying the same modalities of administration (such as early and prolonged administration over time) may be effective. Future studies, adequately addressing this purpose, will be able to give us an answer to this hypothesis. 

## 7. Conclusions 

The ischemic/reperfusion myocardial injury negatively impacts on STEMI outcomes after successful pPCI. The oxidative burst [71] determined by an increased ROS production and reduced GSH availability act as the main players of this phenomenon [9,29,65]. The scavenging activity of GSH maintains thiol groups of enzymes and other proteins in their reduced state, thus preventing cell membrane lipid peroxidation and limiting cardiomyocytes loss [72]. The effects of GSH supplementation are promising in IRI setting, not only based on laboratory findings, such as the observed reduction of the oxidative stress burden [65], but also on clinical ones, such as the decrease in length of hospital stay [66] and the reduced incidence of the adverse left ventricle remodeling at follow-up [29]. Administration in combination with other antioxidants with proven scavenging activity could be hopeful and call for further research on the topic. 

## Data Availability

No new data were created or analyzed in this study. Data sharing is not applicable to this article.
